# Unforeseen Hodgkin Lymphoma Hidden behind Hypercalcemia

**DOI:** 10.1155/2019/4129349

**Published:** 2019-11-18

**Authors:** Moutaz Ghrewati, Balraj Singh, Parminder Kaur, Michael Maroules

**Affiliations:** ^1^Internal Medicine Department at St. Joseph's University Medical Center, 703 Main St., Paterson, NJ 07503, USA; ^2^Hematology-Oncology Department at St. Joseph's University Medical Center, 703 Main St., Paterson, NJ 07503, USA; ^3^Hematology-Oncology Fellowship at St. Joseph's University Medical Center, 703 Main St., Paterson, NJ 07503, USA

## Abstract

As one of the leading causes of hypercalcemia, malignancy is an essential consideration when patients present with such symptomology. While certain tumors are more known to cause hypercalcemia, it can occur with almost any type of cancer. Hodgkin lymphoma is an infrequent cause of hypercalcemia. Only a few case reports have been published in the literature to date. We report an unusual case of a 61-year-old female who presented with altered mental status, nausea, vomiting, and dizziness and was found to have multiple enlarged lymph nodes, and blood work showed hypercalcemia. Biopsy of the cervical lymph node showed Hodgkin lymphoma of the nodular sclerosis subtype. Our case aims to raise awareness of this rare but significant presentation of hypercalcemia and how timely treatment of calcium levels can facilitate the employment of available chemotherapeutic options.

## 1. Introduction

Hypercalcemia is a common paraneoplastic syndrome encountered in oncology practice, constituting up to 30% of patients with cancers [1]. Elevated calcium and resultant symptomology are often the initial triggers for investigation. Hypercalcemia is an adverse prognostic factor in malignancy. While hypercalcemia is more frequent with certain tumors than others, it could be encountered in various types of malignancies. We are reporting a unique case of hypercalcemia associated with Hodgkin lymphoma.

## 2. Case Report

We present a 61-year-old female with a past medical history of diabetes mellitus, hypertension, stroke, and hypothyroidism who presented with altered mental status, nausea, multiple episodes of nonbloody, nonbilious vomiting, and dizziness. Review of systems was positive for intermittent night sweat, diffuse body aches, and unintentional weight loss of 10 pounds over three months. The patient denied any fever, chills, runny nose, sore throat, cough, or shortness of breath. The patient also denied excessive consumption of milk products or antacid or any recent travel or sick contacts. However, reviewing the medications list showed that the patient was on vitamin D supplements with the last use being three months prior to the current presentation.

On admission, vital signs were normal. Physical examination showed dry oral mucosa and multiple enlarged lymph nodes involving the left anterior cervical area, right axilla, and right inguinal area. Nodes were painless, mobile, hard in texture, and measured 2-3 cm in diameter.

The initial laboratory data are revealed in Table 1.

Apart from microcytic anemia and monocytosis, initial blood work results were significant for hypercalcemia of 16.5 (see Table 1). Corrected calcium for albumin using Payne's formula was 16.7 mg/dl [2].

Blood work also showed a prerenal pattern of acute kidney injury (BUN/*creatinine* *ratio* > 20) and hypochloremic metabolic alkalosis, which is attributable to the diuresis effect of calcium. The patient was started on calcium-lowering measures—0.9% normal saline, calcitonin, and bisphosphonate infusion. In the meantime, investigating for the cause of hypercalcemia revealed the results as represented in Table 2.

The patient's presentation and laboratory results (Tables 1and 2) raised the suspicion of lymphoma for which a biopsy of the lymph node and imaging were warranted.

Computed tomography (CT) imaging of the chest, abdomen, and pelvis revealed the involvement of additional lymph nodes in the mediastinum and abdomen. An excisional biopsy of the left cervical lymph node was done. The pathology report showed that the lymph node architecture was entirely effaced by a nodular proliferation of large atypical lymphoid cells with irregular nuclear contour, prominent eosinophilic nucleoli; some are multinucleated and consistent with Reed-Sternberg cell. Immunostains are positive for CD30, CD15, and MUM and weakly positive for Pax-5. These findings are consistent with the classic Hodgkin lymphoma of the nodular sclerosis subtype. The patient was started on ABVD (adriamycin, bleomycin, vinblastine, and dacarbazine) and is in complete remission after two cycles of chemotherapy.

## 3. Discussion

Calcium is an essential mineral component in our body. It is involved in many crucial biological processes, including, but not limited to, vascular contraction and dilation, muscle functions, nerve transmission, and intracellular signaling [3]. Having that pivotal role signifies the importance of maintaining steady levels of calcium in the human body. Main regulatory electrolytes and hormones involved in calcium hemostasis are parathyroid hormone (PTH), calcitonin, vitamin D, and phosphate [4, 5]

After being produced by the parathyroid glands, PTH activates the osteoclasts in the bones to increase bone resorption. PTH also works on the kidneys to increase calcium absorption, urinary phosphate excretion, and the production of the active form of vitamin D by upgrading the activity of renal 1-*α*-hydroxylase. The net target of these processes is increasing serum calcium levels [4, 5].

Pathological causes of hypercalcemia can be classified based on levels of PTH, either PTH-dependent hypercalcemia or non-PTH-dependent hypercalcemia. Parathyroid gland adenoma and adenocarcinomas form the vast majority of PTH-dependent hypercalcemia causes, which is characterized by elevated levels of PTH. On the other hand, non-PTH-dependent hypercalcemia is characterized by normal or low levels of PTH. Among the potential causes of non-PTH-dependent hypercalcemia are hypervitaminosis D, neoplasms, and granulomatous diseases [6].

In our reported case, the patient had a PTH level on the lower limit of the normal, which necessitates further workup to look for non-PTH-related causes of hypercalcemia. While the former medications list included vitamin D supplements (50,000 IU/week), the patient denied any current use of vitamin D supplement or any over-the-counter vitamins. Therefore, having the 25-hydroxyvitamin D level on the higher side of the normal could partially be responsible for our patient's presentation, but it will not be the sole reason for the hypercalcemia. Additionally, coexisting symptomology of nonintentional weight loss and night sweats entailed the appraisal for underlying malignancy.

Several pathways have been reported to explain hypercalcemia associated with malignancies. 80% of these cases are caused by parathyroid hormone-related protein (PTHrP), which explains the importance of testing PTHrP whenever a malignancy is suspected. Most of the remaining 20% results from either elevated levels of activated vitamin D (1,25-dihydroxyvitamin D) due to excess extrarenal activity of 1*α*-hydroxylase or other mediators, such as osteoclastic activating factor (OAF) seen in multiple myeloma, that further enhance bone resorption [1, 7].

In our presented case, the disproportionate elevation of 1,25-dihydroxyviamitn D compared to 25-hydroxyvitamin D employed the excess 1*α*-hydroxylase activity as the main reason for this patient's hypercalcemia (see Table 2). This pathway is entitled to <1% of malignancy-associated hypercalcemia, with non-Hodgkin lymphoma almost always serving the underlying malignancy [8].

Based on pathologic findings, lymphoma is classified into non-Hodgkin lymphoma (NHL) and Hodgkin lymphoma (HL). Hodgkin type is further subclassified into nodular lymphocyte-predominant Hodgkin lymphoma and classical Hodgkin lymphoma. The latter has four different subtypes including nodular sclerosis, lymphocyte-rich, mixed cellularity, and lymphocyte-depleted [9, 10].

Only a few cases have been reported regarding hypercalcemia in Hodgkin lymphoma. To our knowledge, only one-third of these cases is associated with nodular sclerosis subtype [11]. This rare finding further reinforces the rarity and uniqueness of our reported case, and it consolidates the importance of keeping Hodgkin lymphoma among the differentials for 1*α*-hydroxylase-induced hypercalcemia.

Treatment of malignancy-associated hypercalcemia is centered on two main principles: the first is lowering calcium levels, and the second is the treatment of the causative malignancy. The urgency and aggressiveness of decreasing calcium levels are guided by both the serum calcium level and, more importantly, the presenting symptomology. Strategies to achieve this purpose include administration of intravenous fluids with or without loop diuretics, bisphosphonates, calcitonin, and to a lesser extent: gallium nitrate and corticosteroids [12]. However, these measures are considered more palliative and have no mortality benefit. Therefore, it is imperative to start chemotherapy as soon as possible [8, 13].

The presence of hypercalcemia in a patient with malignancy signifies a very poor prognosis, and almost 50% of these patients reportedly die within 30 days [13]. To our knowledge, limited research had shed light on the prognostic significance of hypercalcemia in Hodgkin and non-Hodgkin lymphoma. However, we hypothesize that controlling hypercalcemia would prevent kidney failure and keep the door open for chemotherapy options, which in turn could be translated into mortality benefit.

## 4. Conclusion

Hypercalcemia is commonly encountered in association with malignancies. Cancers can induce hypercalcemia via different pathophysiologic pathways. The increased extrarenal activity of 1*α*-hydroxylase is the primary mechanism in lymphoma. While it is not uncommon to observe hypercalcemia with non-Hodgkin lymphoma, it is scarcely found with Hodgkin lymphoma. Correcting hypercalcemia has a high priority during the initial course of treatment, due to its impact on short-term survival rates. However, no data suggested an impact on long-term prognosis [7]. Therefore, it is imperative to start chemotherapy as soon as possible to achieve the most durable response. We recommend that further research should shed light on the importance of controlling calcium levels, as it may have mortality benefits and allow the employment of different chemotherapy regimens.

## Figures and Tables

**Table 1:** Initial blood work results.

Name of test	Reading	Reference range
White blood cells	7.5K/mm^3^	4.5–11
Hemoglobin	10.7 g/dl	12–16
Hematocrit	33%	36–42
Platelets	266K/mm^3^	140–440
Mean corpuscular volume	77.4 fL	80–100
Red cell distribution width	17.8%	0.5-16.5
Neutrophils	79%	36–75
Lymphocytes	8%	24–44
Monocytes	12%	4–10
Eosinophil	1%	0–5
Basophil	0%	0–2
Sodium	137 Meq/L	135–145
Potassium	4.4 Meq/L	3.5–5
Chloride	97 Meq/L	98–107
Bicarbonate	32 Meq/L	21–31
Blood glucose	125 mg/dl	70–105
Blood urea nitrogen	41 mg/dl	7–23
Creatinine	1.31 mg/dl	0.60–1.30
Calcium	16.5 mg/dl	8.6–10.3
Total protein	6.7 g/dl	6.4–8.4
Albumin	3.7 g/dl	3.5–5.7
Alkaline phosphatase	126 IU/L	34–104
Aspartate aminotransferase	10 U/L	13–39
Alanine aminotransferase	3 U/L	7–25
Lactate dehydrogenase	151 U/L	140–271

**Table 2:** Results for laboratory workup for hypercalcemia in malignancy.

Name of test	Reading	Reference range
PTH^∗^	14.1 pg/ml	11.1–79.5
25-Hydroxyvitamin D	73 ng/ml	30–100
1,25-Dihydroxyvitamin D	143 pg/ml	19.9–79.3
PTHrP^¶^	<2.0 Pmol/L	<2.0

^∗^PTH: parathyroid hormone. ^¶^PTHrP: parathyroid hormone-related protein.
